# History of working conditions and the risk of old-age dependency: a nationwide Swedish register-based study

**DOI:** 10.1177/14034948231188999

**Published:** 2023-08-03

**Authors:** Charlotta Nilsen, Janne Agerholm, Susanne Kelfve, Jonas W. Wastesson, Ingemar Kåreholt, Kirsten Nabe-Nielsen, Bettina Meinow

**Affiliations:** 1 Stress Research Institute, Stockholm University, Sweden; 2 Aging Research Center, Karolinska Institutet/Stockholm University, Sweden; 3 Institute of Gerontology, School of Health and Welfare, Jönköping University, Sweden; 4 Department of Global Public Health, Division of Social Medicine, Karolinska Institutet, Sweden; 5 Department of Culture and Society, Division Ageing and Social Change & Division of Social Work, Linköping University, Sweden; 6 Department of Medical Epidemiology and Biostatistics, Karolinska Institutet, Sweden; 7 The National Research Center for the Working Environment, Denmark; 8 Department of Public Health, Section of Social Medicine, University of Copenhagen, Denmark; 9 Stockholm Gerontology Research Center, Sweden

**Keywords:** Older age, later life, dependency, long-term care, physical working conditions, psychosocial working conditions, work-related stress, longitudinal, Sweden

## Abstract

**Aims:**

There is substantial evidence that previous working conditions influence post-retirement health, yet little is known about previous working conditions’ association with old-age dependency. We examined job strain, hazardous and physical demands across working life, in relation to the risk of entering old-age dependency of care.

**Methods:**

Individually linked nationwide Swedish registers were used to identify people aged 70+ who were not receiving long-term care (residential care or homecare) at baseline (January 2014). Register information on job titles between the years 1970 and 2010 was linked with a job exposure matrix of working conditions. Random effects growth curve models were used to calculate intra-individual trajectories of working conditions. Cox regression models with age as the timescale (adjusted for living situation, educational attainment, country of birth, and sex) were conducted to estimate hazard ratios for entering old-age dependency during the 24 months of follow-up (*n* = 931,819).

**Results:**

Having initial adverse working conditions followed by an accumulation throughout working life encompassed the highest risk of entering old-age dependency across the categories (job strain: HR 1.23, 95% CI 1.19–1.27; physical demands: HR 1.36, 95% CI 1.31–1.40, and hazardous work: HR 1.35, 95% CI 1.30–1.40). Initially high physical demands or hazardous work followed by a stable trajectory, or initially low-level physical demand or hazardous work followed by an accumulation throughout working life also encompassed a higher risk of dependency.

**Conclusions:**

**A history of adverse working conditions increased the risk of old-age dependency. Reducing the accumulation of adverse working conditions across the working life may contribute to postponing old-age dependency.**

## Background

Previous working conditions influence different domains of post-retirement health, including cognitive and physical health [1–3]. However, it is unclear whether working conditions also affect the possibility of living independently in old age. Although many people live healthy into advanced age, towards the end of life most people will experience a period when they need help with activities of daily living and may have to increasingly rely on long-term care (LTC) [4,5]. In this study, we used nationwide register data on LTC to approximate the period with disability in older adults [5,6]. This will yield new insights into how working life shapes old-age dependency in a large and unselected population.

Dependency in old age is multifactorial and can be a consequence of social, physical and cognitive exposures [7]. In Sweden’s universal, and largely tax-financed, social care system, use of social services has been employed to approximate old-age dependency [5]. LTC is available for all inhabitants aged 65+, whose need for support with personal care and/or household chores has been approved by a municipal needs assessor, that is, LTC is needs-tested, not means-tested [8]. More than 80% of community-dwelling people aged 65+, with substantial care needs, that is, being dependent on help with personal activities of daily living (PADLs), such as dressing, eating, and using the toilet, used LTC to some extent [9]. Therefore, in the Swedish context, the use of LTC due to dependency in PADLs may be a valid measure of severe levels of dependency.

The accumulation hypothesis within life course theory suggests that the duration of exposure to a risk factor affects the risk of ill health later in life [10]. Ferraro and Morton [11] define accumulation as a process of amassing one or more things within a phenomenon of interest, in this paper defined as various types of working conditions. Consequently, people who have experienced severe adversity and stress in their lives would be more susceptible to early mortality and illness in adulthood, especially in older age [12]. For example, job strain has been associated with physical impairment, cognitive impairment, and dementia in later life [1, 3, 13–18]. There is also evidence suggesting that adverse physical working conditions, such as physically demanding or hazardous work, have a negative impact on post-retirement health and function [2, 19,20]. However, a limitation common to most research on the long-term impact of working life on late-life health is that it has tended to take a rather static view, or short-run dynamic, of occupation by relying on measures of working conditions assessed only once, such as at midlife or from self-reported longest held occupation [e.g. 13, 17, 19]. Using occupational history data, Zhuo et al. [18] concluded that the duration of job strain influenced cognitive decline in later life [18]. Using survey data, Nilsen et al. [15, 20] found that accumulated stressful and physically demanding occupations across working life had a negative impact on physical and cognitive ageing. However, few studies have had the possibility of capturing working condition trajectories throughout the life course in nationwide register data.

As life expectancy continues to increase, it is important to identify factors that influence old-age disability. Following Ferraro and Morton [11], we defined trajectories of accumulation and de-accumulation of different working conditions to investigate whether a history of adverse working conditions increases the risk of entering old-age dependency within a 2-year follow-up starting from the age of 70+ years. We hypothesised that an accumulation throughout working life from high levels of adverse working conditions is most harmful to independence among older adults.

## Methods

### Data and study population

Using the Total Population Register, we identified people 70 years or older (*n* = 2,016,058). Linking the data with the Swedish Social Service Register (SSSR), we found that 1,053,846 individuals were living independently (without LTC) 2 months prior to the start of follow-up on 1 January, 2014. After excluding municipalities with poor coverage of the SSSR (*n* = 26), the sample size was reduced by 65,727 (*n* = 988,119). Information on occupation was then extracted from the Swedish Population and Housing Census (SPHC) and from the Swedish Occupational Register. A total of 49,935 individuals were excluded at this point since they had no occupational data. Due to missing data on selected covariates (*n* = 6,365), our analytic sample consisted of 931,819 community-dwelling participants in January 2014. The follow-up time was 24 months. Ethical approval for record linkage of Swedish register data was obtained from the Stockholm Regional Ethical Review Board (Dnr 2016/1001-31/4) and the Swedish Ethical Review Authority (Dnr 2020-05700).

### Variables

*Old-age dependency in care* was operationalised as LTC use, that is, living in residential care or receiving homecare assistance with PADLs or >24 h/month of homecare for at least 3 months [6], identified through the SSSR. An LTC duration of less than 3 months could be a sign of short-term dependency, for example, due to an accident, and was therefore not considered to be old-age dependency. The definition of dependency in PADLs was based either on the monthly granted number of hours of homecare or the type of homecare granted, depending on the municipalities’ reporting routines [21]. Moving to residential care was defined as dependency from the first month. Since residential care is only approved if additional homecare would be insufficient, all individuals living in residential care can be assumed to need help with PADLs.

#### Occupational history

Register information on occupation was available from the SPHC at multiple time points (years 1970, 1975, 1980, 1985 and 1990). For the younger cohorts in the sample, additional data were added from the Swedish Occupational Register, which has collated data annually since 2001. Following a similar procedure as with SPHC, occupational data were added for the years 2001, 2005 and 2010. In the case of missing data relating to job title in the SPHC, data from the preceding year then the year after were used. A total of eight measurement points of occupational data were collected, and on average 5.5 occupations were reported per individual (Table I).

**Table I.** Number of individuals in each wave of occupational data.

Wave	Number of individuals (%)
1970	661,125 (70.9)
1975	796,014 (85.4)
1980	833,885 (89.5)
1985	809,700 (86.9)
1990	752,243 (80.7)
2001	420,319 (45.1)
2005	482,766 (51.8)
2010	513,555 (55.1)
Total number of observations	5,269,607
Average reported occupations	5.5

#### Job exposure matrix: working conditions

The available occupations were according to SSYK (*svensk yrkesklassificering*), which is a Swedish version of the International Standard Classification of Occupations, ISCO-88 [22]. The SSYK codes were then matched to a job exposure matrix to assess job strain, and physically demanding and physically hazardous working conditions [23]. The original job exposure matrix was constructed based on the average scores of working conditions for 262 occupations from the 1977 and 1979 Swedish Survey of Living Conditions (*n* = 12,084). In this matrix, gender-specific scores were generated for job control (12 questions), job demands (two questions), physically demanding occupations (five questions), and hazardous occupations (seven questions) (Table II). All scores in the matrix consisted of a linear composite that ranged from 0 to 10. High strain occupations were identified, based on the job demand-control model [24],as the ratio of job demands to job control. For a more comprehensive description of the matrix, see Johnson and Stewart [23].

**Table II.** Description of items included to assess working conditions.

Dimension	Components
Job hazards	Noise Heavy shaking or vibrations Cold Drafts Inadequate ventilation Bad lightning Gas, mist or smoke
Physical job demands	Unsuitable working postures Heavy lifting Work safety Physical exertion Dirt
Work control	Planning of work Planning of vacations Planning of work breaks Selection of supervisors Selection of co-workers Setting of the work pace How time is used in work If there are varied work procedures If there is varied task content Flexible working hours Possibility to learn new things Experience of personal fulfilment on the job
Psychological job demands	If the job is hectic If the job is psychologically demanding

#### Lifetime trajectories of working conditions

Trajectories of life-course exposure to adverse working conditions were calculated for each domain separately. The trajectories were constructed using random effects growth curve models to calculate within-person change in working conditions across the working life. Growth curve models handle missing data by giving more weight to individuals with the most time points. Random effects allow for variation between participants in the individual slope and intercept. Following the method previously used by Nilsen et al., [15, 20] the intercept was divided into low and high via a median split. The slope was divided into decreasing, stable, and increasing trajectories. A slope was considered as decreasing if there was a decrease (i.e. an estimate below zero) and as increasing if there was an increase of more than half a standard deviation above zero. A slope between zero and half a standard deviation increase was considered a stable slope. The slope and intercept were combined to create six trajectory categories: starting low and stable; starting low and decreasing (de-accumulation); starting low with accumulation; starting high with de-accumulation; starting high and stable; or starting high with accumulation. Resulting in six potential trajectories for each working condition domain.

#### Covariates

*Cohabitation status* (cohabiting, living alone), *age*, *sex*, and *country of birth* (Sweden/outside of Sweden) were assessed using the Total Population Register from 2013. *Level of education* was classified as compulsory/upper secondary/tertiary and was assessed using the Swedish Register of Education.

### Statistical analyses

Cox regression models with age as the timescale, with time (age) left-censored at age of entering the follow-up period, were conducted to estimate hazard ratios (HRs) and confidence intervals (CIs) for entering old-age dependency during the 24 months of follow-up. With age as the timescale, time 0 is birth. In this study, the subjects entered the risk set at the age they were at baseline (left-censoring), given that they had reached this point without the event. This means that they were only considered to be at risk when they were actually under follow-up in our study. For example, by using this left-censoring, at age 80, only the subjects who were under follow-up at age 80 were in the risk set when the HR was calculated. Age was chosen as the timescale since we expected the hazard to change more as a function of age than as a function of time-on-study. With left-censored age as the timescale, the results could be interpreted as the risk of earlier dependency. The observations were right-censored either when a participant died before entering old-age dependency or the study ended before old-age dependency occurred (91% of the observations were right-censored). A total of 36,248 died during follow-up. The analyses were adjusted for sex, cohabitation status, country of birth, and level of education at baseline. All statistical analyses were performed with STATA 15.

## Results

Individuals were, on average, 77 years old at baseline, and 53% were women. About 9% initiated LTC during the follow-up of 24 months (Table III). Individuals who entered old-age dependency were on average older, had lower levels of education, were more likely to be women, and to be living alone. The incidence of old-age dependency was similar in people born in Sweden and outside Sweden. The differences in the unadjusted proportions of old-age dependency between trajectories of working conditions were small (Table IV).

**Table III.** Descriptive statistics.

	Old-age dependency during the study period		
	Yes	No	Total
	(*n* = 82,702)	(*n* = 849,117)	(*n* = 931,819)
Age			
Mean (range)	83 (70–102)	77 (70–102)	77 (70–102)
SD	6.2	5.5	5.8
	*n* (%)^a^	*n* (%)^a^	*n* (%)^b^
Sex			
Men	33,060 (7.5)	407,213 (92.5)	440,273 (47.2)
Women	49,642 (10.1)	441,904 (89.9)	491,546 (52.8)
Level of education			
Compulsory	42,207 (10.8)	347,736 (89.2)	389,943 (41.9)
Upper secondary	28,174 (8.0)	322,323 (92.0)	350,497 (37.6)
Tertiary	12,321 (6.4)	179,058 (93.6)	191,379 (20.5)
Cohabitation status			
Living alone	53,659 (12.6)	370,861 (87.4)	424,520 (45.6)
Cohabiting	29,043 (5.7)	478,256 (94.3)	507,299 (54.4)
Country of birth			
Sweden	75.448 (8.9)	769,779 (91.1)	845,227 (90.7)
Outside of Sweden	7254 (8.4)	79,338 (91.6)	86,592 (9.3)

a Row percentage. ^b^Column percentage.

**Table IV.** Descriptive statistics of trajectories of working conditions.

	Old-age dependency during the study period	
	Yes (*n* = 82,702)	No (*n* = 849,117)
Job strain	*n* (%)	*n* (%)
Low/de-accumulation	19,230 (8.1)	219,184 (91.9)
Low/stable	13,575 (10.5)	115,227 (89.5)
Low/accumulation	8152 (8.3)	90,713 (91.8)
High/de-accumulation	30,703 (9.4)	295,718 (90.6)
High/stable	7060 (7.6)	85,783 (92.4)
High/accumulation	3982 (8.6)	42,492 (91.4)
Physical demanding		
Low/de-accumulation	25,007 (7.8)	297,810 (92.3)
Low/stable	8692 (11.6)	66,318 (88.4)
Low/accumulation	4744 (6.7)	66,505 (93.3)
High/de-accumulation	33,913 (10.0)	305,771 (90.0)
High/stable	5953 (8.7)	62,790 (91.3)
High/accumulation	4393 (8.1)	49,923 (91.9)
Hazardous		
Low/de-accumulation	32,066 (9.1)	321,054 (90.9)
Low/stable	7383 (9.8)	67,914 (90.2)
Low/accumulation	2551 (6.5)	36,488 (93.5)
High/de-accumulation	34,941 (9.0)	353,583 (91.0)
High/stable	2441 (8.1)	27,683 (91.9)
High/accumulation	3320 (7.3)	42,395 (92.7)

In the adjusted model, individuals with initially low job strain followed by a stable trajectory had a lower risk of entering old-age dependency during the follow-up (HR 0.78, 95% CI 0.76–0.80) compared with the reference group (i.e. initially low job strain followed by a de-accumulation across working life). Having high job strain followed by a de-accumulation throughout working life also had a lower risk of entering old-age dependency (HR 0.91, 95% CI 0.89–0.92). However, initially high job strain followed by a stable trajectory (HR 1.13, 95% CI 1.10–1.16) or an accumulation throughout working life (HR 1.23, 95% CI 1.19–1.27) increased the risk of entering old-age dependency. Having initially low job strain followed by an accumulation throughout working life was not associated with an increased risk of earlier dependency (Table V).

**Table V.** Risk of old-age dependency.

*N* = 931,819	Old-age dependency	
	Crude model	Adjusted model
Job strain	Hazard ratio (95% CI)	Hazard ratio (95% CI)
Low/de-accumulation	1.00	1.00
Low/stable	0.78 (0.76–0.80)	0.78 (0.76–0.80)
Low/accumulation	1.00 (0.98–1.03)	0.99 (0.97–1.02)
High/de-accumulation	0.92 (0.90–0.93)	0.91 (0.89–0.92)
High/stable	1.14 (1.11–1.17)	1.13 (1.10–1.16)
High/accumulation	1.27 (1.22–1.31)	1.23 (1.19–1.27)
Physical demanding		
Low/de-accumulation	1.00	1.00
Low/stable	0.74 (0.72–0.75)	0.73 (0.72–0.75)
Low/accumulation	1.13 (1.10–1.17)	1.18 (1.15–1.22)
High/de-accumulation	1.01 (0.99–1.02)	1.07 (1.05–1.08)
High/stable	1.23 (1.19–1.26)	1.29 (1.26–1.33)
High/accumulation	1.26 (1.22–1.30)	1.36 (1.31–1.40)
Hazardous		
Low/de-accumulation	1.00	1.00
Low/stable	0.91 (0.89–0.93)	0.94 (0.92–0.96)
Low/accumulation	1.23 (1.18–1.28)	1.32 (1.26–1.37)
High/de-accumulation	0.96 (0.95–0.98)	1.06 (1.04–1.08)
High/stable	1.12 (1.07–1.16)	1.22 (1.17–1.27)
High/accumulation	1.18 (1.14–1.23)	1.35 (1.30–1.40)

*Note*: CI: confidence interval.

Adjusted for living situation, educational attainment, country of birth and sex.

In the adjusted model, an initially low starting point of physically demanding work followed by a stable trajectory across working life had a lower risk of old-age dependency (HR 0.73, 95% CI 0.72–0.75) compared with the reference group. However, having initially low physically demanding work followed by an accumulation throughout working life increased the risk of old-age dependency (HR 1.18, 95% CI 1.15–1.22). Having initially high physically demanding work followed by a de-accumulation (HR 1.07, 95% CI 1.05–1.08) or a stable trajectory (HR 1.29, 95% CI 1.26–1.33) or an accumulation throughout working life (HR 1.36, 95% CI 1.31–1.40) increased the risk of entering old-age dependency (Table V). In the adjusted model, having initial hazardous work followed by a stable trajectory had a lower risk of old-age dependency (HR 0.94, 95% CI 0.92–0.96) compared with the reference group. Having initially low hazardous work followed by an accumulation throughout working life increased the risk of old-age dependency (HR 1.32, 95% CI 1.26–1.37). Having initial hazardous work followed by a de-accumulation (HR 1.06, 95% CI 1.04–1.08) or a stable trajectory (HR 1.22, 95% CI 1.17–1.27) or an accumulation throughout working life (HR 1.35, 95% CI 1.30–1.40) increased the risk of old-age dependency (Table V).

## Discussion

This nationwide longitudinal study investigated working conditions across life and the risk of entering old-age dependency. Experiencing a history of stressful, hazardous or physically demanding jobs increased the risk of entering old-age dependency, particularly continuous exposure to adverse working conditions. With this study, we can confirm earlier findings of long-term exposures from working conditions and their influence on late-life health and function based on smaller more selected samples [15, 17,18, 20] in a large nationwide register-based study.

LTC among older adults is predicted by a person’s ability to care for themselves [7]. There is strong evidence that job strain and physically challenging and hazardous occupations affect both physical and cognitive impairment in later life [13–20]. The results from this study confirmed this in nationwide register data with old-age dependency as the outcome. The similar findings across work domains may be explained by the multifactorial nature of old-age dependency [7]. As hypothesised, we found that having initially high job strain, hazardous or physically demanding work followed by a stable trajectory (i.e. remaining in the same type of occupation) or an accumulation throughout working life (e.g. changing to an occupation that entailed an even higher level of damaging exposure) to be especially harmful. To be exposed to an accumulation of physically demanding and hazardous occupations across working life has previously been associated with lower levels of successful ageing, when assessing mobility and cognitive ageing, and leisure and social engagement [20]. Likewise, having a history of stressful occupations has been associated with physical and cognitive limitations in later life [15, 18].

The accumulation and de-accumulation of different exposures throughout the life course are central to understanding the process of ageing [11]. Following Ferraro and Morton [11], accumulation is reflected in the recurrence of a particular event, that is, the length of time the exposure occurs. Experiencing increasing levels of unsuitable working postures, similar repeated movements, or heavy physical work, harm the physical body during working life and have, for example, been shown to be related to bodily pain and reduced physical function in retirees [2, 13, 19]. Neck- and back pain have been shown to be highly prevalent among workers [25] and are leading causes of disability in high-income countries [26]. Moreover, when stress becomes chronic, it can produce long-term effects through biochemical pathways, such as raised levels of cortisol and insulin. The brain regions involved in cognition and memory can be reached by cortisol with negative long-term effects [27]. Chronic stress can also cause damage to muscles, the heart, the vascular system, and the brain via physiological dysregulation that disturbs metabolism, hormone production, blood pressure regulation, and immune function [28]. Physical frailty in the course of the ageing process, such as chronic illness and the loss of physiologic organ reserve with age [12] may further exacerbate the adverse processes following accumulated exposure to job strain and adverse physical working conditions [29]. However, the timing of the accumulation (i.e. onset) [11], seems to have less of an impact on old-age dependency, regardless of the subsequent trajectory. This suggests that old-age dependency could be delayed by changing from a stressful or physically adverse working environment even in later life. In the case of de-accumulation [11], we found initially high job strain followed by a de-accumulation across working life to be associated with a lower risk of old-age dependency as compared to having initially low job strain followed by a de-accumulation. This may indicate that those with greater initial job strain changed their occupation, either as a result of illness due to chronic stress or voluntarily.

### Strengths and limitations

A strength of this study was that it took advantage of the opportunity to investigate the study aim based on nationwide Swedish registers, providing register-based information about almost a million individuals in our analytic sample. However, our results should be interpreted with some caution. First, although LTC is needs-tested (i.e. high sensitivity), not all individuals who need assistance in daily life apply for LTC. However, we tried to minimise the impact of informal care on the use of LTC by focusing on LTC due to dependency in PADLs. Most individuals who face such a high level of dependency have used LTC to some extent [4,5], implying an acceptable specificity of our outcome measure. Therefore, we consider the use of LTC due to dependency in PADLs to be a reasonable proxy for old-age dependency at a population level. The use of LTC is also determined by factors other than individual health-related needs, for example, thresholds for the allocation of public services and access to informal care. Men are more likely than women to live with a spouse until death, implying that men may experience a shorter period requiring LTC than women as they more often have access to informal care from a spouse [5]. There may also be cultural differences that influence to what extent an individual seeks formal care. To minimise such bias, the analyses were adjusted for sex, cohabitation status and country of birth. Furthermore, compared with activities that focus on domestic chores, the use of LTC due to severe disability is less affected by individual characteristics. Second, 24 months of follow-up could be considered a relatively brief period in which to investigate the risk of entering old-age dependency, as the mean age of the sample was 77 years when the LTC data were collected, and the mean age of entering old-age dependency was 83. If followed for a longer period, it is expected that more individuals would have entered old-age dependency during the study period. However, whether a longer follow-up would yield other findings is unknown, as this would depend on the patterns in the incidence of old-age dependency between those exposed and unexposed. We did not apply a longer follow-up as the LTC data were limited prior to 2013. Third, using an average population-based matrix does not consider inter-individual variations within occupations. However, the advantage of using this matrix is that it is relatively free of bias caused by individual reporting differences, and the matrix has also been shown to have good internal validity [22]. Finally, although this study presents a unique opportunity to validate previous findings in a large nationwide register-based study, a limitation with register data is the lack of many relevant confounders.

## Conclusions

In this study, we found that having a history of stressful, hazardous or physically demanding jobs increased the risk of entering old-age dependency, thus supporting the accumulation risk model within life course theory. From a policy perspective, it is important to find potentially modifiable predictors of old-age dependency to be able to develop strategies and interventions to prevent or delay disability in later life. This study’s results indicated that promoting a healthy workplace by reducing the accumulation of physically demanding, hazardous and stressful jobs across the working life, may prevent or delay old-age dependency.
